# Artificial Intelligence-Assisted Diagnostic Cytology and Genomic Testing for Hematologic Disorders

**DOI:** 10.3390/cells12131755

**Published:** 2023-06-30

**Authors:** Lealem Gedefaw, Chia-Fei Liu, Rosalina Ka Ling Ip, Hing-Fung Tse, Martin Ho Yin Yeung, Shea Ping Yip, Chien-Ling Huang

**Affiliations:** 1 Department of Health Technology and Informatics, The Hong Kong Polytechnic University, Hong Kong, China; lealem.bimerew@connect.polyu.hk (L.G.); chia-fei.liu@polyu.edu.hk (C.-F.L.); martin-ho-yin.yeung@polyu.edu.hk (M.H.Y.Y.); 2 Department of Pathology, Pamela Youde Nethersole Eastern Hospital, Hong Kong, China; ikl482@ha.org.hk (R.K.L.I.); thf963@ha.org.hk (H.-F.T.)

**Keywords:** artificial intelligence, hematologic disorders, diagnostic cytology, genomic testing, machine learning

## Abstract

Artificial intelligence (AI) is a rapidly evolving field of computer science that involves the development of computational programs that can mimic human intelligence. In particular, machine learning and deep learning models have enabled the identification and grouping of patterns within data, leading to the development of AI systems that have been applied in various areas of hematology, including digital pathology, alpha thalassemia patient screening, cytogenetics, immunophenotyping, and sequencing. These AI-assisted methods have shown promise in improving diagnostic accuracy and efficiency, identifying novel biomarkers, and predicting treatment outcomes. However, limitations such as limited databases, lack of validation and standardization, systematic errors, and bias prevent AI from completely replacing manual diagnosis in hematology. In addition, the processing of large amounts of patient data and personal information by AI poses potential data privacy issues, necessitating the development of regulations to evaluate AI systems and address ethical concerns in clinical AI systems. Nonetheless, with continued research and development, AI has the potential to revolutionize the field of hematology and improve patient outcomes. To fully realize this potential, however, the challenges facing AI in hematology must be addressed and overcome.

## 1. Introduction

Artificial intelligence (AI) refers to computer programs that contain robust datasets and simulate human intelligence, including problem-solving skills and learning [1]. The concept of AI has been around since the early 1950s and, in recent years, AI has become an increasingly integral part of our daily lives, with applications in medicine to store, retrieve, and uncover patient data. The emergence of global pandemics, chronic diseases, and big data requires the use of AI-assisted technologies. Medical image analysis, clinical decision making, and patient behavior analysis are some of the applications of AI in healthcare settings [2]. The use of AI in diagnostic hematology is one of the most promising applications in medicine [3]. In the field of hematology, AI has been used since 1994 when three knowledge-based systems were implemented in Europe to analyze complete blood counts (CBCs), immunophenotyping, and bone marrow reports in leukemia patients [4,5].

Machine learning (ML) is a subset of AI that enables computers to learn without any preset rules. It is performed by utilizing algorithms developed to process the dataset to automatically detect patterns and perform tasks, such as detection and classification, in various domains [6]. Furthermore, ML can be classified into three types. These are supervised ML, unsupervised ML, and reinforcement ML [1]. Supervised ML is used to predict outcomes based on a labeled training set while unsupervised ML identifies patterns or groupings within data. Reinforcement ML is a combination of supervised and unsupervised learning, which maximizes accuracy by trial and error. However, ML systems require a significant amount of data to learn effectively [1].

Deep learning (DL) is a powerful subset of ML which refers to a multilayered network of artificial neurons aimed at creating models from raw data. The expanding amount of data provided by wearables, smartphones, and other mobile monitoring sensors can be handled by DL algorithms in a number of medical fields. The algorithms are separated into layers to create an artificial neural network (ANN) which functions similarly to the human brain [7]. ANNs consists of three major layers, including the input layer, the middle-hidden layer(s), and the output layer. Each hidden layer increases the complexity of the learned features, and ANNs can perform various tasks such as image recognition, pattern recognition, classification, translation, and medical diagnosis. There are several DL-based techniques, including multi-layer perception (MLP), recurrent neural network (RNN), and convolution neural network (CNN) [7]. MLP is the simplest type of neural network, in which ANNs are layered sequentially and information flows through the system unidirectionally. RNNs analyze input data one by one while simultaneously storing all memories of encountered inputs, making them useful for interpreting sequential information such as deoxyribonucleic acid (DNA) sequences. CNNs learn common features and perform spatial correlations from captured images. They ranks hierarchies, and the output data are converted into an input signal for the following learning steps [8].

These AI ML and DL models are the foundation of AI systems applied in hematology. In recent years, researchers have utilized these models to develop algorithms for automated interpretation of blood cell morphology, classification of blood disorders, prediction of disease prognosis, and identification of new biomarkers. For instance, automated blood cell morphology analysis using DL models has shown promising results in reducing inter-observer variability and improving diagnostic accuracy [9]. Moreover, ML algorithms have been employed to predict the risk of thrombosis in patients with myeloproliferative neoplasms (MPNs) and to identify new subtypes of leukemia based on genomic data [10]. These models have also been applied to the analysis of large datasets of hematology images, such as peripheral blood smears (PBSs), bone marrow aspirates (BMAs), and lymph node biopsies, and have produced AI-aided diagnosis systems that are able to assist pathologists to make more accurate diagnoses [11].

By leveraging the power of AI and ML models, researchers are making significant progress in the field of hematology. Given the increased prevalence of hematologic disorders and emerging global pandemics, these models have the potential to revolutionize hematology by providing faster and more accurate diagnoses, improving patient prognosis, and lowering healthcare costs. However, there are still challenges that need to be addressed, such as the need for large datasets and the potential for bias in the training data. Therefore, further research and development are necessary to fully realize the potential of AI in hematology. The most recent updates from well-known electronic databases, including PubMed, the Web of Science, Medline, and Google Scholar, and search engines for gray literature such as Google, were meticulously combed through. Conference abstracts were excluded while we considered full-text, peer-reviewed publications, books, review papers, and preprints pertinent to the area and written in English. This review highlights the current updates and challenges in the role of AI in diagnosing hematologic disorders, risk stratification, prediction of disease prognosis, challenges, and future directions in implementing AI in the health system.

## 2. Current Applications of AI in Hematologic Cytology

Non-communicable diseases are becoming a global health problem, resulting in the death of 41 million people each year and accounting for 74% of all deaths globally [12]. Cancer, a complex disease caused by multiple factors, is the world’s second leading cause of death, particularly in developing countries [13]. Hematologic malignancies, also known as blood cancers, are a set of neoplastic disorders marked by persistent abnormal cell growth in the bone marrow, lymph nodes, and/or blood [14]. These complex diseases require experts for detection, diagnosis, risk identification, and treatment. This approach is becoming a challenge as healthcare facilities are overburdened due to the increased prevalence of the disease and the emergence of global pandemics, implying the need for an accurate and efficient patient management system. Despite the emergence of cutting-edge diagnostic technologies, manual expert evaluation remains indispensable for disease reclassification, validation, and result interpretation.

Current diagnosis of hematologic malignancies is based on evaluating patient blood parameters, biopsies to test small pieces of tissue for cancerous cells, and imaging tests to rule out metastasis of specific disease conditions. The treatment options may include any combination of chemotherapy, bone marrow transplantation, immunotherapy, radiation therapy, targeted therapy, and transplantation [15]. The emergence of malignancies with multiple drug resistance and their atypical nature put a double burden on the detection and diagnosis of hematologic disorders. A more efficient, accurate, and traceable approach is mandatory to tackle this emerging public health concern. AI is an emerging approach that can help to analyze big data, identify patterns, correlate with clinical diagnosis, and predict patient survival in healthcare settings. AI can be applied in various hematologic diagnostic services, including in CBCs, cellular morphology analysis, detection of cellular inclusions, identification and quantification of malignant cells, molecular characterization, and prediction of patient prognosis.

### 2.1. Review of Blood/Marrow Smears

Review of peripheral blood smears (PBSs) and bone marrow smears (BMSs) is a powerful diagnostic tool that provides rapid, reliable information about a variety of disorders such as leukemia, anemia, infections, and allergies [16]. Smear review in bone marrow examination is frequently used for diagnosing infiltrated hematologic disorders and helps differentiate between the underlying causes. PBS and BMS examinations are essential to confirm a clinically suspected disease and to uncover a previously unsuspected diagnosis [17]. Depending on the circumstances, peripheral blood, BMAs, and trephine biopsy are possible samples to use for analyzing the cytomorphology of blood cells as well as their pattern of distribution [18]. However, the routine microscopic examination of blood and marrow smears in the hematology laboratory takes a longer time, is cumbersome and error-prone, and is inefficient in high-patient-flow healthcare settings. Given the dynamic nature of hematologic disorders and atypical features of various cells, AI and ML approaches would provide a more rapid, accurate, and efficient examination of PBSs and BMSs. Furthermore, it would reduce turnaround time for patient diagnosis, reduce interpersonal variation, improve healthcare delivery, and predict the prognosis potential of patients.

The use of AI in hematology is still limited, with only a few Food and Drug Administration (FDA)-approved AI devices currently available on the market. These devices are primarily used for the examination of PBS morphology. Among them, CellaVision and Morphogo are two representative systems used for the examination of PBS and BMA slides, respectively. CellaVision is an automated digital image analyzer that has been approved by the FDA [19,20]. It can be connected to a hematologic analyzer and slide-making device to perform a CBC, blood film preparation, staining, cell location, and identification automatically. The system includes a high-quality microscope and a digital camera connected to a computer. When a PBS is scanned, the analyzer identifies the monolayer of cells, and the image analysis software captures cell images for pre-classification.

**Figure 1:**
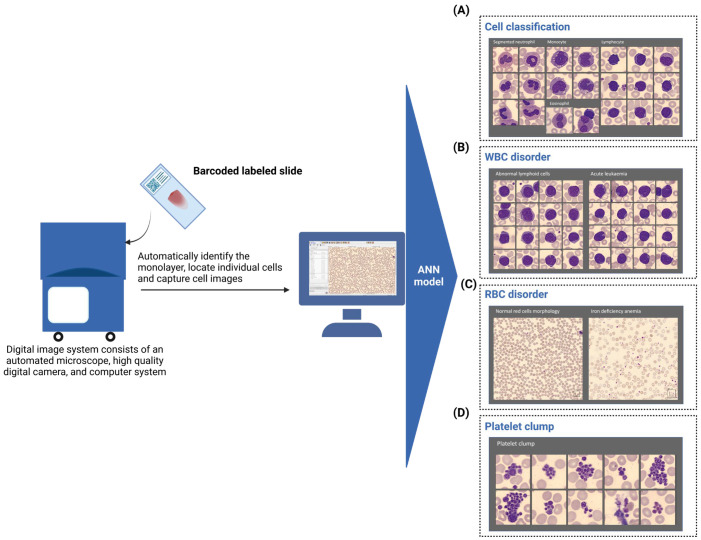
The process of classifying abnormal cells using CellaVision’s ANNs. (**A**) Normal blood cell morphology. The system detects and classifies normal mature WBCs, RBCs, platelets, and immature cells. (**B**) WBC disorder. The system detects abnormal WBCs, such as increased blast cells, and suggests acute leukemia. (**C**) RBC disorder. The system characterizes RBC size, color, and shape and suggests iron-deficiency anemia. (**D**) Platelet clumps. The system detects and classifies platelets, including platelet clumps. The automatically captured images are pre-classified as different cell types by a pre-processing algorithm and then fed into an ANN model for further classification into subtypes such as “abnormal WBCs”. The ANNs are trained on a large dataset of labeled images to learn the features that differentiate each subtype, allowing for accurate and efficient classification of abnormal cells. ANN, artificial neural networks; RBC, red blood cell; WBC, white blood cell.

The pre-classification of both white blood cells (WBCs) and red blood cells (RBCs) is achieved by means of ML-based ANNs, which are a collection of connected artificial neurons (nodes) organized into hidden layers that can perform different transformations based on the inputs received. Signals are transmitted from the input to the output layer, possibly passing through the hidden layers multiple times [21]. CellaVision’s ANNs are well trained using a large database of images. Individual WBCs are captured by a 100× oil-immersion lens, and pre-classification is performed for the WBC differential count, allowing for the classification of up to 17 cell types, including immature cells such as blasts and myelocytes. The pre-classification of WBCs is based on cell color, special features, geometry, and texture [22]. The captured image of the WBCs is compared to the database in the library, and the results can be verified by users (Figure 1). The pre-classification of WBCs has been reported to have an overall accuracy of approximately 98%, showing a good correlation with the manual identification of cells [23]. CellaVision (DM96) with ANN support demonstrated 100% sensitivity and 94% specificity in identifying blasts, as well as 100% sensitivity and 97% specificity for typical WBCs and nucleated RBCs. The system also has extra features such as the ability to consult and review slides remotely and perform cell-by-cell quality control [24].

RBCs are pre-characterized by a program called CellaVision Advanced RBC application, which captures them using a 50× oil-immersion lens. RBCs are classified based on morphological abnormalities such as size, central pallor, roundness, border notching, and inclusion bodies [19]. They can be pre-classified into 21 morphological categories, and RBC grading can be reported. According to reports, the sensitivity of CellaVision ranges from 33% for RBC agglutination to 100% for detecting poikilocytosis (sickle cells, stomatocytes, etc.). The specificity was 84.5% and 99.5% for schistocyte and sickle cells, respectively [25]. In addition, CellaVision helps to characterize platelets in peripheral blood [23].

The potential of AI in hematologic diagnosis is undeniable because it offers several benefits, such as reduced turnaround time and improved consistency in patient results due to easier standardization. CellaVision can enhance the training efficiency of staff, as well as reduce eyestrain by allowing laboratory technologists to review and verify results on a computer screen. However, this technology has limitations that prevent it from fully replacing traditional slide reviews by manual microscopy. CellaVision has low sensitivity and specificity in identifying lymphoblasts and plasma cells, which share similar morphological features with lymphocytes. In cases of leukemia, CellaVision can identify blast cells, but cannot differentiate myeloblasts, lymphoblasts, and monoblasts. Additionally, CellaVision has poor sensitivity and specificity in identifying schistocytes, which are crucial for the diagnosis of microangiopathic hemolytic anemia (MAHA). According to the guidelines of the International Council for Standardization in Hematology, even the presence of <1% schistocytes in a PBS should be reported for the diagnosis of thrombotic thrombocytopenic purpura and hemolytic uremic syndrome [19]. However, the morphological definition of schistocytes has not been standardized, and there is high variability in identifying them among technologists. Moreover, CellaVision cannot identify malaria parasites and hemoglobin H (Hb-H) inclusion bodies as it only captures monolayer cell images and has low resolution [26]. Finally, CellaVision faces difficulty in identifying neonatal blood cells. Billard et al. reported that the performance of CellaVision had the lowest accuracy in classifying neonatal samples (81%), which might be due to their increased fragility [27].

**Figure 2:**
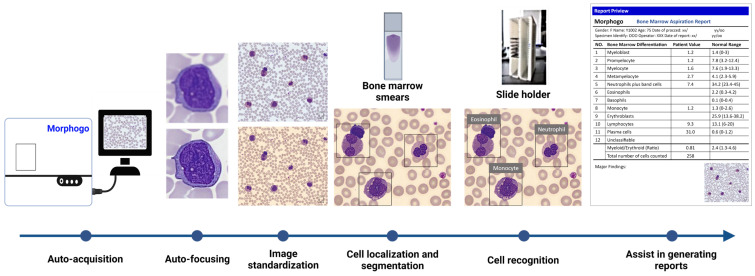
The workflow of the Morphogo system. Clear cell images are automatically obtained and standardized. Following this, the nucleated cells in the images are located, segmented, and identified. Finally, further cell classification and quantification are auto-analyzed and auto-calculated, respectively, using the CNN model.

BMS examination plays a crucial role in diagnosing hematologic diseases. Although manual microscopy remains the gold standard, it is time consuming and can be subject to bias among hematologists. Morphogo is an AI-aided BMA smear analysis system that can differentiate nucleated cells into specific categories and provide information useful for diagnosis. It employs a 27-layered CNN that captures multiple high-resolution images of the BMS and pieces them together to generate a full picture of the target morphology (Figure 2). The convolution filter of the CNN can enhance the quality of the cell cluster image by removing or emphasizing specific cell characteristics during image processing, such as sharpening, blurring, and edge detection [28]. Cancer cell cluster features are extracted and classified as carcinoma or non-carcinoma [29]. Fu et al. demonstrated the performance of Morphogo in identifying hematologic lineage cells in 230 cases and showed a classification accuracy of 85.7–91% [20,30]. The system’s average sensitivity and specificity were found to be 69.4 and 97.2%, respectively [20]. Another study by Lin et al. reported that Morphogo demonstrated an accuracy of 82% and specificity of 91% in identifying metastatic cancer cells when compared to pathologists, with a reliability coefficient of 0.827 [22]. Consequently, it is thought that Morphogo has potential as an AI tool for BMS analysis in the future, possibly negating the need for additional analyses such as flow cytometry and molecular analysis.

On the other hand, the Morphogo AI-based system was able to diagnose technically challenging metastatic non-hematopoietic tumor cells in BMSs. For instance, the method can classify metastatic cancer cells with 82.2% accuracy and 91.3% specificity. The study indicated that Morphogo could be a reliable method (reliability coefficient of 0.827) in the detection and classification of cells of cancer cell clusters (area under the curve (AUC): 0.865) [29]. Furthermore, recent advances in AI technologies enabled Morphogo to detect megakaryocytes from bone marrow samples. Want et al. used bone marrow digital images with CNNs to identify and characterize megakaryocytes with a sensitivity and specificity of 96.6% and 89.7%, respectively [31].

Scopio is a newly FDA-approved high-throughput hematology digital cell morphology platform using a browser-based application. This advanced computational photography imaging tool with AI support enables the capture of digital scans with a full field of view of the monolayer and a feathered edge at a 100× oil-immersion magnification level. The system can hold up to 30 slides and process up to 40 samples within an hour, which can fulfill the high-throughput requirements of large hospitals and labs. It can pre-classify WBCs, estimate platelets, and evaluate the morphology of RBCs. According to a study by Katz et al. in 2021 [32], the Scopio full-field PBS (Labs X100) (Scopio, Israel) has an accuracy of 96.3%, a sensitivity of 87.9%, and a specificity of 97.6% when compared to the manual blood smear assessment. This new technology was tested with 335 patients’ normal and abnormal PBSs. Additionally, they examined RBC morphology and found that the test results had a 99.8% agreement with the reference methods. Similar to this, the platelet estimation by Scopio Labs X100 full-field PBS had a 94.9% accuracy, a 90% sensitivity, and a 96.3% specificity [32]. The system was updated to incorporate a BMS assessment with Scopio’s unique AI-powered full-field BMA (FF-BMA) application to obtain a digitized BMA image including a nucleated differential count, myeloid to erythroid ratio, megakaryocyte count, maturation stages, and morphology assessment [33].

Another AI-assisted blood cell analysis system, Mantiscope, uses AI to fully classify blood cells and find abnormalities. A scanner and the cloud are two components of the in vitro diagnostics system. The blood smear is automatically digitized using the scanner. Following this, the system analyzes the smear samples using AI after uploading the images to the cloud based on the patient’s barcode. Medical professionals can edit the AI recommendations using the system’s annotation interface. Additionally, the system can count, identify, and pre-classify cells in BMAs [34].

Vision Hema provides automatic analysis and pre-classification of blood cells derived from a PBS. The system analyzes WBCs, RBCs, platelets, and reticulocytes in great detail. It also analyzes pathological changes in cells such as those with degenerative changes, blasts, atypical lymphocytes, erythroblasts, smudge cells, and other non-WBC cells. Modules for cell identification and pre-classification in bone marrow samples, body fluids, and cervical cytology are also available [35].

It was a challenge to detect atypical and dysmorphic cells in patients with hematologic disorders, such as myelodysplastic syndromes (MDS), where their diagnosis is based on morphological findings in peripheral blood and bone marrow, particularly with other conditions presenting similar phenomena such as aplastic anemia (AA). Kimura et al. developed a novel AI-supported image analysis platform based on deep convolutional neural networks and CNNs, which can assist in differentiating between MDS and AA [36]. They evaluated their method using a dataset consisting of 695,030 images taken from 3261 PBSs and were able to differentiate blood cells according to their morphological features with a specificity and sensitivity of 96% and 93.5%, respectively. The automated MDS diagnostic system was able to differentiate MDS from AA with 96.2% sensitivity and 100% specificity (AUC: 0.99) [36].

Similarly, the use of DL helps to identify subtypes of acute myeloid leukemia (AML). Acute promyelocytic leukemia (APL) is a subclass of AML and is characterized by a translocation of the retinoic acid receptor alpha gene located on chromosome 17. Bleeding and thrombosis are major causes of fatal complications and death in patients with APL [37,38]. A multistage DL that automatically reads images of BMSs, accurately segments cells, and subsequently predicts APL is used to automatically detect and predict APL from BMSs. This DL method showed an average detection precision of 97% and differentiated APL from other AML subtypes and healthy individuals [39]. Li et al. on the other hand, were able to differentiate human diffuse large B-cell lymphoma (DLBCL) and non-DLBCL with 100% accuracy using multiple CNNs to classify pathologic images [40]. A study undertaken on BMSs to identify and classify bone marrow cells showed that the convolution and attention network model (CoAtNet), a hybrid of CNNs and transformer models, showed the best performance (accuracy >95%) when compared to other models evaluated in a similar fashion [41]. AI-assisted BMS evaluation helps to predict mutations in hematologic malignancies. Eckardt et al. used a multi-stage CNN-based prediction model to diagnose AML and predict *nucleophosmin 1* mutation status from a BMS (AUC: 0.92) [42].

Diagnosis of lymphoma based on clinico-morphological features is challenging due to the heterogeneity of the malignant cells, time-consuming process, and requirement of expertise. Integration of AI-based models, such as CNNs, in the differential diagnosis of lymphoma helps to deliver a high standard of care and further improve the therapy of lymphoma patients. Automatic classification of common lymphoma types was improved via the use of pattern detection mechanisms based on the content of each image [43]. Efficient Net CNNs help to classify non-Hodgkin’s lymphoma (NHL) cases with high accuracy (95.6%) from histologic images [44]. Miyoshi et al. reported a DL method for detecting and classifying malignancy lymphoma. They collected whole-slide images (WSIs), stained with hematoxylin and eosin (H&E) from BCL, follicular cell lymphoma (FCL), and lymphoid hyperplasia patients. The method is able to accurately classify the three types of lymphoma cases with an accuracy of 97%, which is higher than the average accuracy (76%) recoded by the pathologist using WSIs [45]. An ML-based diagnosis helps to classify T-cell lymphomas. Yu et al. collected 40 histological WSIs for dataset training in a DNN. They succeeded in extracting picture characteristics and categorizing T-cell lymphomas, such as intestinal T-cell lymphoma, which pose difficulties in morphologic diagnosis [46].

Similarly, a DL-based CNN approach helps to diagnose lymphoma from cytochemical-stained smears. They collected H&E-stained slides from 128 cases. Using WSIs, they established a diagnostic model for the four lymphoma groups, including benign lymph node, DLBCL, Burkitt lymphoma, and small lymphocytic lymphoma. Accordingly, they could predict the different types of lymphoma from H&E-stained images with an accuracy of 95% using the CNN model [47]. Syrykh et al. employed a CNN-based approach called Bayesian neural networks (BNN) to distinguish FCL from follicular hyperplasia. They used H&E-stained lymph node slides and were able to detect FCL with an overall accuracy of 91% [48]. AI models were also used to detect and classify rare lymphoma types such as mucosa-associated lymphoma tissue (MALT). Pezoulas et al. developed federated AI algorithms such as federated gradient boosting trees (FGBT), FGBT with dropouts (FDART), federated multilayer perceptron (FMLP), and federated multinomial naïve bayes (FMNB). Among these, the FDART was able to identify and classify MALT lymphoma in primary Sjogren’s syndrome patients with an accuracy of 82.8% [49].

The ML-based method LymphoML can predict lymphoma subtypes from H&E-stained slides. The new method helps to predict the different types of lymphoma, including DLBCL (F1 score: 78.7%), classic Hodgkin lymphoma (F1 score: 74.5%), and mantle cell lymphoma (MCL) (F1 score: 71.0%), where the F1 score is a combined measure of both specificity and sensitivity. Considering the nuclear shape features, the method performed better with a diagnostic accuracy of more than 85% [50].

Immunohistochemical staining (IHC), a more advanced type of histopathology, is an immunologic technique that uses antigen–antibody interactions to detect cellular or tissue antigens under a microscope [51]. The technique uses various antibody panels and requires experienced pathologists to identify hematolymphoid diseases. Studies indicated the integration of ML with IHC in the pathologic diagnosis of hematologic neoplasms. ImmunoGenius, an ML-based mobile application, helps to predict various lymphoma types using IHC results. Abdul-Ghafar et al. reported that validation of the ImmunoGenius on 3052 cases of lymphoid neoplasms was able to predict and differentiate lymphoma with an accuracy of 91.8% [52]. Accordingly, the method showed a decrease in error rate and convenience after external validation and could be applied in clinical practice. The potential limitation of this algorithm is that it does not take into account the clinicopathology of patients, which needs to be considered in the future development of AI-based diagnosis using IHC in hematologic neoplasms [52]. ML provides accurate differentiation of DLBCL based on IHC compared to gene expression profiles. Cost et al. reported that ML helps to differentiate germinal center and non-germinal center subtypes of DLBCL with an accuracy of 91.6% [53]. Similarly, Carreras et al. applied AI-based ANNs to identify prognostic markers for MCL. They analyzed 123 cases and used ANNs to identify 58 genes that predicted MCL patient survival with high accuracy (AUC: 0.9). Among prognostic markers, based on IHC, they revealed that the regulator of G-protein signaling 1 (RGS1) was associated with poor survival [54]. Due to the lack of single clinical features for hematolymphoma, labor-intensive diagnostic approaches in tissue staining and examination, and the need for multiple panels of antibodies, integration of ML in clinical diagnostics could improve the delivery of care and prognosis of patients. AI-based algorithms are limited regarding lymphoma diagnosis, differentiation, risk stratification, and identification of prognostic markers. There is currently no FDA-approved, ML-based algorithm for lymphoma diagnosis. In order to develop a more precise ML-based diagnostic algorithm that would be affordable and improve health care delivery, more research on large-scale databases, including rare disorders, should also be evaluated.

### 2.2. Detection of Hb-H Inclusion Bodies

Anemia can cause a variety of physical symptoms as well as visual signs, making it difficult to diagnose. As a result, a novel AI approach based on ML as a model is recommended for diagnosing, selecting treatments, and predicting prognosis [55]. Thalassemia is a group of inherited genetic disorders associated with a defect in hemoglobin (Hb) synthesis. Studies showing the role of AI in detecting and classifying thalassemia have been reported [56]. Detection of Hb-H inclusion bodies in RBCs is a crucial test for the diagnosis of α-thalassemia and is commonly performed in hematology laboratories using supravital stains such as brilliant cresyl blue (BCB) or new methylene blue. However, the manual examination of stained cells under a microscope is a laborious and time-consuming process, with one case taking up to 15 min [57]. Moreover, the possibility of human error during screening may result in the omission of Hb-H-positive cells, particularly in cases where Hb-H-positive cells are very low [58]. Therefore, there is a need for a more efficient and accurate method of detecting Hb-H inclusions in RBCs.

Lee and colleagues developed an AI-based protocol for the detection of Hb-H inclusions that promises to improve the accuracy and efficiency of screening [59]. The protocol involves capturing digital images of BCB-stained blood smears at various magnifications and inputting them into an AI model that has been trained to recognize Hb-H-positive cells. The training process involves classifying cells as either Hb-H-positive or -negative and setting up the ground truth for the AI’s decision-making process. Once the ground truth is established, the AI can analyze test images and assign a prediction confidence score (PCT) to each cell image, indicating the level of confidence that its prediction is correct. Lee et al. reported that the AI system ML-CNN achieved a sensitivity of 90.9%, a specificity of 99%, and an accuracy of 97.6% in Hb-H inclusion screening when the PCT threshold is set at 0.2 or higher. This represents a significant improvement over manual screening, which is labor intensive, time consuming, and prone to human error, particularly in cases with low levels of Hb-H-positive cells [59].

In order to further clarify the previous sentence, it is crucial to point out that the low PCT of the AI model in Hb-H inclusion detection may not necessarily point to a problem with the model itself but rather with the type of input data used. Kensert et al. suggested that the high similarity in image patterns between Hb-H-positive and -negative cells might pose a challenge for the AI model to accurately classify the cells [60]. This highlights the importance of continually refining and optimizing the training dataset for the AI model to improve its accuracy and reduce false negatives. Additionally, it may be beneficial to incorporate other features and information beyond image analysis, such as clinical history and laboratory results, to further enhance the accuracy of Hb-H inclusion detection.

Aside from its use in advancing morphology-based diagnosis, ML can also be applied to other Hb variants, such as Hb-S and Hb-D, which require precise diagnosis and prediction in the diagnosis of hemoglobinopathies. These Hb variants can be identified using high-performance liquid chromatography (HPLC); however, Hb-S and Hb-D cannot always be reliably distinguished from one another due to differences in the software used for gradient programs and HPLC models. The use of ML, such as ANNs, helps to classify and predict Hb variants. Ucucu et al. demonstrated ANN-based recognition of human Hb variants in the HPLC system. The study used clinical samples of known hemoglobinopathies and found that ML was able to detect Hb variants with an accuracy of 99%, specificity of 99%, and sensitivity of 99% [61]. This implies that an ANN-based ML method has demonstrated high performance and has the potential to be integrated into diagnostic service as a tool for the detection and prediction of hemoglobinopathies.

### 2.3. Flow Cytometric Analysis

Flow cytometry is a powerful laboratory assay for the detection, diagnosis, and monitoring of various hematologic disorders such as leukemia and lymphoma [62]. While several markers have been developed for flow cytometry analysis in recent years, manual interpretation of results, including cell population gating, remains necessary. The technical procedures in handling flow cytometry data and prolonged gating time required to deliver patient results are the existing gaps in the field. To overcome this limitation, researchers introduced AI applications to flow cytometric analysis. The use of AI-supported techniques in flow cytometry has been shown to improve workflow and provide accurate and timely results. For example, researchers developed an AI-based algorithm to classify patients with chronic lymphocytic leukemia (CLL) based on their flow cytometric profiles. Salama et al. developed deep neural networks (DNN) to diagnose CLL. They evaluated the hybrid DNN approach on treated CLL patient samples using a 10-color panel to detect minimal residual disease (MRD) in CLL and compared it with expert analysis. Accordingly, the hybrid DNN approach showed a reduction in gating time to 12 s per case from 15 min per case when compared to the manual process, yielding an overall accuracy of 97.1% [63]. Relapse is common in acute leukemia, particularly in B-cell acute lymphoblastic leukemia (ALL). Identification of potential markers indicating the prognosis during diagnosis is a challenge. Chulian et al. demonstrated using AI-based ML flow cytometry analysis to identify potential prognostic markers in 56 pediatric B-cell ALL patients. Based on Fisher’s linear discriminant for relapse prediction, they identified cluster of differentiation 38 (CD38) as a potential marker for relapse, indicating that B-cells with low CD38 expression might serve as a potential indicator for relapse in ALL patients [64].

Similarly, Vial and colleagues developed a DL-based method to accurately detect MRD in AML using multiparameter flow cytometry (MFC) data. These studies demonstrate the potential of AI in enhancing the accuracy and efficiency of flow cytometric analysis in identifying MRD in AML. In this novel approach, flow cytometry results are transformed into a self-organized map (SOM) that is then converted into a two-dimensional (2D) format that is accessible by CNNs. The SOM file of the sample is then input into a CNN, which generates a prediction of MRD, implying that the use of AI allows for an easy and robust assessment of AML-MRD patients in the absence of molecular markers [65].

Integration of ML in flow cytometry analysis helps to classify B-cell NHL (B-NHL). Gaidano et al. integrated ML into the available database of 1465 B-NHL samples. Accordingly, they were able to identify nine clinico-pathologic known subtypes of B-NHL with an accuracy of 92.7%, which includes DLBCL, Burkitt lymphoma, FCL, splenic lymphoma, MCL, marginal zone lymphoma (MZL), and lymphoplasmacytic lymphoma (LPL) [66]. Zhao et al., on the other hand, converted MFC raw data into a multicolor 2D image using a SOM. The SOM file of the sample was then input into a CNN, which generated a prediction of the B-NHL subtype. The AI model successfully classified B-NHL subtypes from normal cases with a 0.94 weighted F-score, indicating high accuracy. The model successfully differentiated B-NHL from lymphocytic leukemia, MZL, MCL, prolymphocytic leukemia, and FCL. However, limitations arise when the similarity of flow cytometry results is high as the classification may be wrongly reversed, for example, between LPL and MZL [67]. Further developments are needed to resolve this issue, but this novel AI application in flow cytometric analysis may pave the way for fully automated flow cytometry analysis in the future.

Recent studies explored the use of AI in other areas of flow cytometric analysis beyond disease classification. Studies indicated that an AI-based method for automated gating of cell populations in flow cytometry data achieves high accuracy and consistency across different datasets [68]. In addition, Arvaniti and colleagues developed an AI-based tool called CellCNN to identify rare cell populations in flow cytometry data, which could have significant implications in the diagnosis and monitoring of various diseases [69]. These studies highlight the potential of AI to not only improve disease classification, but also to automate and enhance various aspects of flow cytometric analysis.

Understanding computation data and applying them for patient diagnosis and management is a hurdle in the healthcare setting. DL tools could overcome this limit by interpreting the AI prediction system. The use of the “local interpretable model-agnostic explanations” algorithm for explainable artificial intelligence (XAI) helps to interpret the AI data with an accuracy of 98.4% [70]. AI is becoming increasingly important in clinical decision making, particularly in areas where skilled labor is in short supply. Clinical decision systems are now being incorporated into AI-supported flow cytometry applications as a result of recent advancements in AI technology to support clinical diagnosis, treatment options, and treatment outcome prediction. This will help in determining the best course of treatment for the patient and will improve healthcare delivery. The system replicated expert judgment by employing a number of training procedures based on a baseline database containing various factors and the estimated dynamic impact of each factor [71] (Table 1).

## 3. AI-Assisted Genomic Testing for Hematologic Disorders

### 3.1. Cytogenetic Karyotyping

Fluorescence in situ hybridization (FISH) has been used for detecting chromosomal abnormality by applying a DNA-targeted probe; however, the existing technique is time consuming and technically demanding. Although the DNA FISH model is the technique of choice to locate the genomic loci in a single allele, it requires user monitoring, which limits managing big data in a short time. The use of AI-integrated, FISH-based diagnosis of chromosomal abnormality will shorten the time required and improve the diagnosis. Gudla et al. developed a more efficient and accurate CNN-based DNA FISH detection method named SpotLearn. Accordingly, this CNN-based method could detect FISH signals with an accuracy greater than 98% [85]. DeepSpot is another ML-based method for the detection of RNA FISH. The method was able to accurately detect RNA FISH signals (accuracy: 97%). However, applying this AI-based method in lymphoma diagnosis needs further investigation [86]. Similarly, FISH was used for risk stratification of MRD patients with plasma cell myeloma. Integrating ANN into the diagnostic algorithm improved the detection and high-risk stratification of myeloma cases with an accuracy of 94% [87].

Cytogenetic karyotyping is a well-established method for detecting chromosome abnormalities in patients. However, the process of preparing and interpreting the results can be time-consuming and requires experienced technologists [88]. To address these challenges, researchers explored the use of AI in cytogenetic karyotyping. For example, AI models have been developed for automatic chromosome segmentation and pairing [89]. Despite these advances, challenges remain in cases where the chromosomes are distorted, overlapping, or blurred, as is often the case in bone marrow samples.

To address these challenges, new AI models were developed such as the ChromoEnhancer model developed by Bokhari and colleagues in 2022 [90]. This model uses an image-to-image translation model called the CycleGAN model and does not require a training set. One key feature of ChromoEnhancer is that it enhances each chromosome image separately, resulting in an output image with similar contrast. In comparison to other models, such as histogram equalization, block-matching, and 3D filtering, ChromoEnhancer provides a clear outline of the chromosome with sharp contrast in the chromosomal bands. Moreover, ChromoEnhancer is able to retain abnormality features in the chromosome, such as deletion and translocation [90]. Thus, the ChromoEnhancer model has the potential to improve the resolution and accuracy of cytogenetic karyotyping analysis for all sample types.

Karyotype analysis from G-banded metaphase images has important clinical significance in the diagnosis, treatment, and prognosis of hematologic tumors. Accordingly, CNNs perform well when it comes to image recognition. The metaphase system was originally used for chromosomal analysis in cells from different tissue types, e.g., lymphocytes and bone marrow. Hu et al. developed an AI-based CNN to detect chromosomal changes automatically. They used Softmax activation function mapping to classify chromosomes using a CNN with multiple layers that was trained using the labeled dataset. The results showed that the CNN-based identification of chromosomes was 93.8% accurate [91]. On the other hand, Chen et al. reported that ML could be applied in chromosome segmentation analysis. They developed a chromosome segmentation model called ChroSegNet based on U-Net, a CNN-based algorithm, which is able to extract key features of chromosomes and provide an accurate result (accuracy: 93.3%) [92].

**Figure 3:**
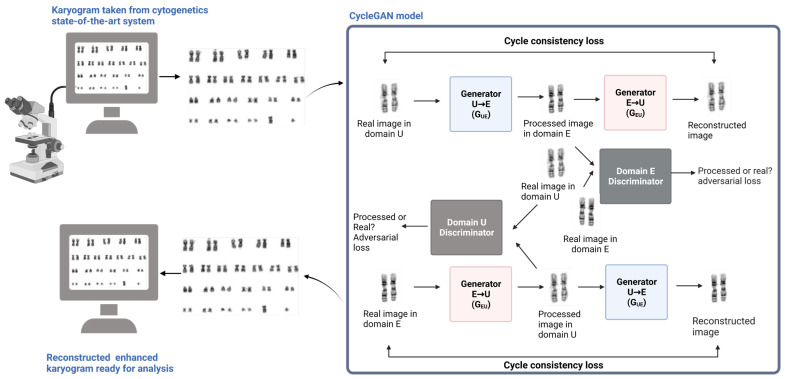
ChromoEnhancer model architecture. The images of karyogram, taken from a cytogenetics state-of-the-art system, are input into the cycleGAN model, then the enhanced karyogram images with improved resolution, sharp contrast, and high accuracy are output for further analysis.

Similarly, DL, such as DNNs, uses advanced algorithms in the field of AI applied for detecting and classifying chromosomal abnormalities. Through advanced AI applications, Ikaros, supported by DNN, was used in separating and classifying banded chromosomes. This system applied fluorescence R-banding to obtain all karyograms from bone marrow and blood samples. The DNN predicted the chromosome class and the required rotation angle from the individual chromosome image. This CNN-based application helped to predict chromosome classes with 98% accuracy and classify chromosome bands as normal and abnormal in hematologic malignancies. It further reduced the processing time by 42% when compared to the conventional karyotyping workflow [93] (Figure 3).

The use of AI in cytogenetic karyotyping holds great promise for improving the accuracy and efficiency of diagnosis. However, more studies are required to address some of the issues with this strategy, including the requirement for large datasets for building AI models and the shortcomings of weak AI classifiers that still require histological classification or medical examination. Overall, the development of advanced AI models, such as ChromoEnhancer, represents an important step toward improving the diagnosis and treatment of hematologic disorders [90,94].

### 3.2. Sequencing for Profiling of Genetic Markers

AI has been applied primarily in image-based diagnosis. However, accurate and precise disease classification is challenging with this approach. To address the difficulties in hematologic malignancies, a comprehensive strategy is necessary. Genetic profiling is crucial for the management of hematologic neoplasms because it provides important data for diagnosis, risk stratification, therapeutic choices, monitoring of residual disease, prognosis, and treatment resistance. Hematologic diseases, especially hematologic malignancies, are characterized by unique gene expression profiles, such as the *BCR-ABL1* (breakpoint cluster region-Abelson 1) fusion gene in chronic myeloid leukemia (CML) and different gene expressions in thalassemia. Therefore, genetic testing, such as sequencing, has become a common diagnostic method [95]. However, analyzing and interpreting the results often requires expertise, which can introduce human error and bias [7,94].

To address this issue, a research team developed a platform that combines a middle-throughput gene expression assay and ML to identify the subtyping of B-NHLs. Using a panel of 137 genetic markers, the gene expression assay classified B-NHLs according to their cellular origin, the composition of their microenvironment, and the configurations of their immunoglobulin genes [94]. The team then developed an ML system, the random forest classifier, to classify cases into seven major subtypes of B-NHLs, such as germinal center B-cell lymphoma and activated B-cell lymphoma. The results showed 80–100% concordance with previous classification results [94].

The conventional classification of leukemia, such as AML, is based on a clinicomorphopathologic classification, which groups AML into primary and secondary AML types. This kind of classification lacks correlation to molecular signatures. ML integrated genomic signatures for AML and was able to identify novel genomic AML subclasses. The model included genomic data from primary AML and secondary AML patients and applied both supervised and unsupervised ML methods. This ML model showed a 97% accuracy in classifying the different subtypes of AML and predicting their prognosis [96].

These studies suggest the possibility of integrating AI into routine clinical testing by combining genetic profiling with limited laboratory resources, such as resources for DNA library construction only. The use of well-trained AI can avoid bias and human error in diagnosis since the classifier achieves a high accuracy of diagnosis based on historical databases. However, the limitations of using AI were uncovered, including the requirement for a larger database, such as data from patients with rare conditions, to enhance the accuracy of diagnosis and avoid missing rare cases. All AI classifiers mentioned in the studies were weak AI, which still requires histological classification or further examination by physicians for some cases. Therefore, the ultimate goal would be to develop a DL diagnostic system that does not require any historical database support or secondary examination [94,97].

### 3.3. Whole-Genome Sequencing for Analysis of Copy Number Variations

Accurate copy number variation (CNV) detection is still a major issue for the community because of the unique characteristics of tumor samples and the complicated nature of tumor genomes. The collection and evaluation of representative data from various regions of the genome is also required for CNV interpretation, making it difficult to provide consistent, high-quality clinical interpretation of CNVs. The present diagnostic strategy employing next-generation sequencing requires excellent-quality data, which is not always the case owing to the lack of standards and the presence of numerous biases. Furthermore, the analysis of big data takes time and affects the quality of care in addition to the data quality. Advances in genomics and AI led to the development of new approaches for the diagnosis and treatment of hematologic disorders. One such approach involves the use of AI to analyze whole-genome sequencing (WGS) data for the detection of CNVs. CNVs are a form of genetic mutation that can contribute to the development and progression of hematologic malignancies. For instance, detecting CNVs in the *ZMAT4* gene had a strong association with hematologic malignancies such as AML [98]. The gain of chromosome 21 in patients with Down Syndrome was associated with leukemogenesis [99], and gain of chromosome 1q was associated with multiple myeloma (MM) [100]. Furthermore, the detection and characterization of CNVs could help in monitoring the prognosis of patients with hematologic malignancies such as ALL [101]. Although WGS improved the efficiency of detecting chromosomal alterations in various hematologic disorders [102], detecting CNVs can be challenging because they can be difficult to distinguish from noise in sequencing data.

A study was published using AI to develop a DL model called AI-CN. AI-CN could accurately detect and classify CNVs in WGS data from patients with hematologic malignancies. Haferesh et al. reported that AI-CN could classify chromosome bands with an accuracy of 98.6% [103]. CNV-P is another AI-based method for the detection of chromosomal alterations. The researchers tested the model on a large cohort of patients and found that it outperformed other CNV detection methods in terms of sensitivity and specificity [104]. These models could help to identify clinically relevant CNVs that are not detected by other methods, demonstrating their potential for improving the accuracy of CNV detection and prediction in hematologic disorders.

In another study, researchers used a combination of WGS and AI to identify CNVs in patients with AML [105]. The researchers developed a DL model called CopyNumberGAN to identify CNVs from the patient sequencing data. They compared the performance of CopyNumberGAN to other CNV detection methods and found that it had higher sensitivity and specificity. The results suggest that AI-assisted CNV detection can improve the accuracy and efficiency of diagnosis and treatment for patients with AML.

These studies demonstrate the potential of AI-assisted WGS for CNV analysis in hematologic disorders. Using AI to analyze sequencing data, researchers can improve the accuracy and efficiency of CNV detection, which could lead to better prediction of patient diagnosis, therapeutic responses, and prognosis. The development of AI algorithms for CNV detection has the potential to revolutionize the field of hematologic disorders, enabling the development of more personalized and effective treatment strategies.

### 3.4. Single-Cell Sequencing Analysis

Single-cell sequencing is a powerful tool that allows researchers to study individual cells within a heterogeneous population, providing valuable insights into complex disease mechanisms at the cellular level [106]. In recent years, there have been several studies utilizing AI-assisted single-cell sequencing analysis for hematologic disorders [107]. For example, an ML model can be used to differentiate patients with hematologic disorders from healthy individuals. More than eight AI-based ML models were tested, and ANN models showed the highest performance (accuracy: 82.8%) in screening hematologic malignancies when compared to other ML models [108]. A study used AI to analyze single-cell RNA sequencing (scRNA-seq) data from patients with CLL. The researchers developed a DL model called scDeepCluster, which identified distinct subpopulations of cells based on gene expression profiles. They found that scDeepCluster outperformed other clustering methods in terms of accuracy and speed and identified novel subpopulations of cells that were associated with disease progression [109,110,111].

Another study used AI to analyze scRNA-seq data from patients with MDS. The researchers developed a DL model called DeepMDS, which was able to accurately predict patient outcomes based on gene expression profiles. They found that DeepMDS outperformed other prediction models in terms of accuracy and identified several novel biomarkers that were associated with disease progression [112].

Additionally, AI can assist in the analysis of single-cell sequencing data from patients with AML. A DL model called support vector machine (SVM) was developed, which identified cell subpopulations and predicted patient outcomes based on gene expression profiles. They found that SVM outperformed other clustering and prediction methods in terms of accuracy and identified several genes that were associated with disease progression [113,114]. Recurrent infections and treatment failures are two common occurrences in the management of hematologic malignancies and must be identified early [115]. For patients with hematologic malignancies, such as AML, AI could assist in selecting the best course of treatment. Potential therapeutic protein targets have been identified based on target analysis, and various AI algorithms were used to rule out therapeutics and select promising therapeutic candidates [116]. On the other hand, AI helps to predict personalized medicine for patients with AML. Gimeno et al. applied the multi-dimensional module optimization (MOM) ML method to help to predict and interpret the appropriate drug for AML patients based on the predicted genetic mutations from RNA-seq data [117]. The effectiveness of these AI-based techniques using scRNA-seq data from patients with hematologic malignancies must be examined in more detail. Overall, the use of AI-assisted, single-cell sequencing analysis has the potential to improve the diagnosis and treatment of hematologic disorders by providing insights into the molecular mechanisms underlying disease progression. By identifying novel subpopulations of cells and biomarkers associated with disease progression, researchers can develop more targeted and personalized therapies for patients [67,109,116].

There are emerging AI-assisted single-cell sequencing platforms that will be furthered via validation using clinical samples from patients with hematologic disorders. A multi-level convolutional neural network (MulCNN) was developed by Jiao and his colleagues to provide a unique, single-cell gene expression profile by extracting critical features through multi-scale convolution while filtering noise [118]. BERMUDA (batch effect removal using deep autoencoders), another novel DL-based method, provides a higher-resolution cellular subtype. It merges a number of batches of scRNA-seq data with heterogeneous cell compositions. Wang and his colleagues indicated that the new model outperformed existing methods for removing batch effects and distinguishing cell types in multiple datasets, including real scRNA-seq datasets [119].

**Figure 4:**
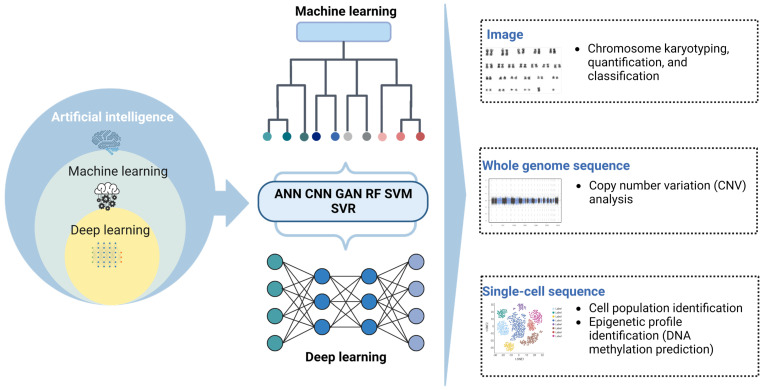
The application of AI in genomic testing for hematologic disorders. AI-based computational methods, including ML and DL, have been developed in hematologic disorders of genomic analysis, such as chromosome karyotyping, copy number variation, cell clustering, and epigenetic profile prediction. ANN, artificial neural network; CNN, convolution neural network; CNV, copy number variation; DNA, deoxyribonucleic acid; GAN, generative adversarial network; RF, random forest; SVM, support vector machine; SVR, support vector regression.

Similarly, reference component analysis 2 (RCA2) uses reference transcriptomes as a guide and adopts graph-based clustering (scalability). It offers user-friendly downstream analysis modules, new mouse and human reference panels, and support for the establishment of custom panels. It also provides cell type-specific quality control for accurate clustering of data from heterogeneous sources. The method was evaluated on single-cell data from human bone marrow and healthy peripheral blood mononuclear cells (PBMCs) [120]. Although there are platforms for AI-assisted single-cell sequencing, analysis, and prediction, more research is needed to assess their effectiveness in clinical settings using samples from patients with hematologic neoplasms (Figure 4 and Table 2).

Due to their capacity to offer solutions for diverse biological samples, scRNA-seq techniques are growing in popularity. Thought should be given to the difficulties with single-cell technologies when using new ML approaches to handle single-cell sequencing data. Limitations in benchmarking analysis tools, integration of data across models, types of cells and measurements, varying levels of resolution, handling of cell sparsity including a high dropout rate leading to the absence of expression, mapping single cells to a reference atlas, generalizing trajectory inferences, dealing with errors, and missing data are a few of the challenges, which impede downstream analysis and influence the performance of emerging ML models in the diagnosis of hematologic neoplasms [121,122]. Therefore, new AI/ML-based systems must take into account current difficulties and offer more precise, comprehensive, and trustworthy prediction and diagnostic algorithms employing representative big scRNA-seq datasets.

### 3.5. Epigenetic Profiling to Identify Novel Biomarkers

Epigenetics is caused by a complex interaction between a person’s genotype and the environment, which plays a role in disease development. Recent studies showed the potential of AI-assisted epigenetic profiling for hematologic disorders. For instance, researchers utilized AI to analyze DNA methylation data from patients with ALL. The developed DL model, called single-cell omics references (EpiScore), accurately predicted patient outcomes based on DNA methylation patterns, outperforming other prediction models. EpiScore also identified novel biomarkers that were associated with disease progression, showing the potential of AI in enhancing our understanding of ALL [123,124].

In another study, a DL model called MethylNet was developed to analyze DNA methylation data from patients with MDS. The AI model accurately classified patients into different subtypes of MDS based on their DNA methylation patterns, outperforming other classification methods. Furthermore, MethylNet identified several novel biomarkers associated with disease progression, suggesting its potential for improving the diagnosis and treatment of MDS [125,126].

MM, a heterogeneous malignant tumor, is distinguished by abnormal plasma cell clonal proliferation in the bone marrow and is often accompanied by apparent monoclonal immunoglobulin protein. DNA methylation analysis in MM patients showed heterogeneity associated with transcriptomic variability, implying the need for more accurate prediction models [127,128]. A study employed AI to analyze chromatin accessibility data from patients with an MM. The researchers developed a DL model called Epimetheus, which accurately predicted patient outcomes based on chromatin accessibility patterns, outperforming other prediction models. Epimetheus also identified several novel biomarkers associated with disease progression, demonstrating the potential of AI-assisted epigenetic profiling for MMs [129,130].

Other epigenetic prediction tools, including DeepCpG [131], are also available, although further research is required to evaluate their performance in predicting the detection and prognosis of hematologic malignancies.

These studies highlight the potential of AI in enhancing our understanding of hematologic disorders by analyzing epigenetic data. By using AI to analyze DNA methylation and chromatin accessibility data, researchers can identify novel biomarkers associated with disease progression, ultimately leading to better diagnosis and treatment for patients.

Although AI-based ML improved diagnostic accuracy and identification of potential biomarkers for epigenetic alterations, there are still limitations that need to be considered to improve the implementation of ML in clinical diagnosis. One potential challenge might arise from the low incidence of cases and increased differentially methylated regions (DMR) within a single case. Further, it has been indicated that epigenetic datasets have more variables than patient samples, limiting the effectiveness of AI-based algorithms. An analysis of a DNA methylation dataset reveals nonlinear relationships in addition to the DMR. Several CpG sites may appear on the same gene, which may influence other regions of the methylome. This suggests that, despite recent advancements in epigenetic research, future developments in AI technologies for detection, risk classification, and prognosis should take into account newly emerging multiple causal factors for epigenetic changes, such as environmental factors, and work on representative large DNA methylation datasets [132].

**Table 2:** AI applications in sequencing for hematologic disorders.

No.	Method/Device	AI Model	Function	Accuracy (%)	Year	References
1	Ikaros	CNN, DNN	Chromosome karyotyping myelofibrosis	98	2023	[80]
2	ChromoEnhancer method	CycleGAN	Bone marrow karyotyping	NA	2022	[90]
3	Softmax	CNN	Chromosome classification	93.8	2019	[91]
4	KaryoNet	MFIM, DAM	Chromosome quantitation and classification	98.4–99.6	2023	[133]
5	CNV-P	DL	CNV detection	90	2021	[104]
6	CUP-AI-Dx:	CNN	Predicts tumor primary site and molecular subtype	98.5	2020	[134]
7	scDCC	DL	Cell clustering	90	2021	[110]
8	DeepCpG:	DL	DNA methylation	NA	2023	[131]
9	EPiScore	DL	DNA methylation	NA	2018	[135]
10	MOM	ML	Therapeutic prediction	NA	2022	[117]
11	MulCNN	CNN	Cell clustering and batch effect removal	NA	2023	[118]
12	BERMUDA	DL	Cell clustering and batch effect removal	NA	2019	[119]
13	RCA2	MEM, SNN	Cell clustering and batch effect removal	NA	2021	[120]
14	SpotLearn	CNN	DNA FISH detection	98	2017	[85]
15	DeepSpot	DNN	RNA FISH detection	97	2022	[86]
16	ChroSegNet	CNN, U-Net	Chromosome segmentation	93.3	2023	[92]

AI, artificial intelligence; BERMUDA, batch effect removal using deep autoencoders; CNN, convolution neural network; CNV, copy number variation; DAM, deep assignment module; DL, deep learning; DNA, deoxy-ribonucleic acid; DNN, deep neural networks; FISH, fluorescence in situ hybridization; MEM, memory efficient fast cluster; MFIM, masked feature interaction module; MoM, multi-dimensional module optimization; MulCNN, multi-level convolutional neural network; NA, not applicable; RCA2, reference component analysis 2; scDCC, single-cell deep constrained clustering; SNN, shared nearest neighbor clustering.

## 4. AI-Assisted Clinical Prediction Models for Hematologic Disorders

Clinical prediction and treatment optimization play crucial roles in clinical diagnosis. Hematologic diseases are linked to the development of complications, putting the patient at risk for secondary conditions and eventually death. The use of AI as a potential tool to perform clinical prediction for hematologic disorders has been explored in recent years [136,137]. For example, a 50-variable random forest model (IAC-50) was developed to predict the overall survival of patients with MMs. The model used various parameters, such as age, first-line treatment, and gene expression, to predict the optimal first-line treatment that would offer the best predicted survival rate for MM patients. However, further validation with larger databases was needed to achieve a more precise predictive value [138]. Another study published recently also used AI to develop a clinical prediction model for MM patients. The researchers analyzed clinical and genetic data from over 2000 MM patients and developed a model that accurately predicted patient outcomes. The model outperformed other prediction models in terms of accuracy and identified several novel biomarkers associated with disease progression [139]. Patients with MM have a higher risk of acquiring bacterial and viral infections due to immune deficiency and other therapeutic effects, which also contribute to death. Different ML prediction models were applied to 564 MM patients to predict the risk of infection. The ML-XGBoost model performed significantly better in terms of prediction than other models (AUC: 0.8664). This type of AI model will assist in lowering the risk of infection and enhancing the prognosis for MM patients [127].

A study used ML algorithms to analyze clinical and genetic data from AML patients. The researchers developed a model that accurately predicted patient outcomes, outperforming other prediction models in terms of accuracy. The model also provided valuable insights into the underlying mechanisms of AML. Furthermore, ML could help to predict the in-hospital mortality of AML patients. A study on a cohort of 29,613 hospitalized AML patients used three ML models, including ML algorithm logistic regression, decision tree, and random forest. Among these, ML logistic regression and random forest models showed better performance in predicting the in-hospital mortality of AML patients (AUC: 0.78) [140]. Identification of MRD cases is a challenge in clinical practice and is time consuming. ML could help to differentiate AML from MDS and healthy individuals. An ML model with over 2000 patient samples showed accurate differentiation of AML from MDS (AUC: 0.943) [141].

Similar to this, a study used AI to create a clinical prediction model for CLL patients. The researchers developed an algorithmic population description approach (ALPODS) based on an XAI model that accurately predicted patient outcomes after analyzing MFC data from samples from more than 150 CLL patients. The ALPODS XAI algorithm model outperformed (AUC: 0.95) other prediction models in terms of accuracy [142]. AI empowered with ML can identify new cases with a high risk of infection. A study developed the CLL treatment-infection model, which can predict the risk of infection for at least two years with a precision of 72% and a recall of 75% [143].

Risk identification and stratification are essential components in clinical diagnosis to predict patient outcomes and reduce therapeutic costs. AI could help to predict risks in chronic hematologic diseases, including thrombosis. Ryan et al. showed that ML methods help to reduce risks in hospitalized patients with deep vein thrombosis [10]. A study on risk stratification and prediction in patients with myelofibrosis (MF) using genetic data showed that ML improved case risk stratification. According to the study, the AIPSS-MF (Artificial intelligence prognostic scoring system for myelofibrosis) model helped to identify patients at risk of secondary MF and predicted survival status [80].

On the other hand, a study by Shanbehzadeh et al. compared eight ML methods to predict the survival status of CML patients with either alive or deceased outcomes. Among the eight ML approaches, the SVM showed the best performance with an accuracy of 85.7%. This implies that AI could help predict long-term patient outcomes and choose the best treatment options [144].

It is important to note that clinical prediction models require regular evaluation and optimization over time and in different settings, since the estimated condition may not always completely represent actual events in the future [137]. Although AI-assisted clinical prediction models are more commonly used in clinical research than in clinical practice, they have the potential to provide better personalized and more effective treatments for patients with hematologic disorders. Applications of AI approaches to integrating transcriptomic, genomic, and epigenetic data would contribute to a deeper comprehension of patient phenomena and aid in diagnosing hematologic neoplasms, including MRDs.

## 5. Challenges in Developing Clinical AI Systems

AI in healthcare environments increases the accuracy of diagnosis, lowers overall healthcare costs, facilitates information sharing, and enhances target treatment, including the treatment of rare diseases. However, developing clinical AI systems presents a unique set of challenges that must be addressed to ensure their accuracy and reliability in clinical practice. One of the main challenges is to ensure the quality and availability of data. AI algorithms are trained on data, and if the data contain bias or are not representative of the patient population, the algorithm may produce inaccurate or biased results. Therefore, it is essential to ensure that the data used to train AI algorithms are of high quality, free of bias, and include variation among patient backgrounds and clinical conditions [1,136]. On the other hand, in some situations where the incidence of hematologic malignancies is rare, representative information may not be acquired to develop AI-based models for prediction, diagnosis, and risk stratification. A possible example could be ocular MALT lymphoma and other lymphomas such as enteropathic T-cell lymphoma. In such conditions, AI fails because of its inability to get the global solution to the problem and tends to get stuck in local minima due to the small sample size.

Another challenge in developing clinical AI systems is to ensure that they are regularly evaluated for accuracy, sensitivity, and specificity. The evaluation process should be undertaken systematically and frequently to identify any issues and to ensure that the AI system remains accurate and reliable over time [1,136]. In addition, the development of XAI has improved the communication between AI and physicians. XAI allows physicians to better understand how AI arrives at its conclusions, which is important for building trust in the system and for ensuring that the results are interpreted correctly [145].

Ethical issues are also a concern when developing clinical AI systems. One of the main ethical concerns is data privacy. AI systems process large amounts of personal and sensitive information, and if this information is not adequately protected, it can pose a significant threat to patient privacy and security. Strict regulations, such as the general data protection regulation, must be applied to ensure the security of health medical records, protect user data, ensure data integrity and traceability, and ensure the security of electronic signatures [146].

Healthcare providers typically own medical records and patient-related information. Sharing clinical data should involve a degree of transparency in patient compliance. The best course of action would be to increase patient awareness of the types of data, the nature of the information in their records, and the recipients of those records. Patients ought to be not only told when their data are used in research but also informed of the findings and potential repercussions of that research. Jessica Morley et al. reviewed the narrations related to AI ethics and suggested that policymakers should consider and take action for AI ethics and help AI improve the quality of care [147].

There is great potential and major concerns over privacy, confidentiality, and control of data in the era of AI about individuals once the data are shared. However, the benefits of data sharing should outweigh the drawbacks and be based on the interest of the larger community. AI applications in the diagnosis of hematologic neoplasms should comply with state- and region-based regulations. It would be in patients’ best interests to be actively involved in the development of policies on data sharing. Owners of AI-based technologies, however, should be aware of the potential risks involved with AI-based data management and processing, both in success stories and in instances when diagnosis went wrong. It also aims to pave the way for public policies to support a balanced agenda that safeguards personal information while enabling the use of data to improve clinical patient care and public health. Furthermore, healthcare policymakers at the global level need to create a unified legal and policy framework that supports a fair agenda that protects individual privacy rights, restricts commercial exploitation, and sends a strong public message while allowing the use of AI in healthcare datamining for both research and commercial purposes. Regulation should prioritize patient autonomy and consent and support ever-evolving data anonymization and protection techniques [148].

The challenges in developing clinical AI systems extend beyond just ensuring the quality of data and regular evaluation of the AI algorithms. Another major challenge is to ensure the interoperability of different AI systems used across various clinical settings. The lack of interoperability can lead to fragmented care and inconsistencies in treatment recommendations [149]. Currently, we do not understand how AI works, and it is usually used as a black box. However, in the last few years, the field of interpretable AI has become more prevalent, and there might be studies on the application of interpretable AI in hematologic disease and cytology. There is a need for standardization in the development and implementation of AI systems in clinical practice. Therefore, the issue of bias in AI systems must be addressed to ensure that the algorithms do not perpetuate existing disparities in healthcare delivery [150,151].

It is also critical to make clinical AI systems user-friendly and accessible to healthcare professionals with various levels of technical specialization. User interfaces should be designed to facilitate easy navigation, and the output of the AI system should be presented in a clear and understandable format to aid decision making and change the future of healthcare services [152]. In order to use AI systems in clinical practice successfully, healthcare providers also need to receive adequate training and support. Furthermore, the application of AI in hematology outside of the realm of research receives little attention in postgraduate medical education and training. This hinders the development of hematology-related AI research and the application of the technology in healthcare systems [153].

## 6. Conclusions

In conclusion, the application of AI in hematology diagnostics is on the rise, and it has the potential to greatly facilitate hematology diagnosis by combining results from different diagnostic methods. The use of AI in hematology diagnostics will help reduce the turnaround time, reduce diagnostic costs, and predict disease outcomes. However, it is important to note that AI cannot fully replace manual diagnosis due to its limitations, including limited databases, lack of validation and standardization, and the risk of systematic errors and bias. Furthermore, the use of AI poses data privacy issues; therefore, regulations on clinical AI systems, including evaluation of AI systems and regulations on ethical issues, are necessary to protect user information and privacy. To address the field’s current challenges, more research must be conducted, and AI should be incorporated into medical education.

## Figures and Tables

**Table 1:** AI-based integrated diagnostic methods in hematologic cytology laboratories.

No.	Methods	Function	Model Used	Accuracy (%)	Year	References
1	WSI analysis	Automated detection of HB-H inclusions in RBCs	ML, CNN	97.6	2021	[59]
2	CellaVision	Blood and marrow smear image analysis Advanced RBC morphology analysis	AI, ANN	98	2020	[19,23]
3	DI-60	Automated cell image analyzer		91	2022	[72]
4	Morphogo	Blood and marrow smear image analysis	AI, CNN	85.7–91	2021	[20,30]
5	Scorpio	Fulfilled PBS and BMA image analysis	AI	94.9	2020	[33]
6	Mantiscope	Digital PBS and BMS preparation and image analysis	ANN, CNN	NA	2018	[73]
7	Vision Hema	Blood cell identification and pre-classification	AI	NA	2019	[35]
8	EasyCell Assistant	Automatic detection and classification of cell morphology	ML	NA	2023	[74]
9	YOLOX-s model	BM cell classification	DNN	92.5	2023	[75]
10	Nextslide	Automated digital imaging	AI	99.7	2012	[76]
11	XGBoost	Differentiate PV, ET, and MF	CNN, DL	90	2021	[77]
12	HematoNet	BM cell detection and classification	DL, CoAtNet	95	2022	[41]
13	Ensemble model	WBC detection	DL	98.8	2023	[78]
14	Automated BMT phenotyping	Morphological identification of megakaryocytes	AI, ML	95	2020	[79]
15	AIPSS-MF	Risk stratification of MF patients	ML, random forest	82	2023	[80]
16	ImageStream (Amnis)	Identification of white blood cells	ML, SVM	99	2020	[81]
17	Attune CytPix	High-resolution, real-time imaging of cells in flow cytometry	AI	NA	2023	[82]
18	ImageStream (Amnis)	Leukemia monitoring	CNN, linear SVM	98.2	2021	[83]
19	AlexNet	Detection of ALL and AML	ML, CNN	98	2022	[84]
20	DNN-FC	Detection of CLL-MRD	AI, DNN	97.1	2022	[63]
21	XAI	Translate AI data in ALL	AI, DL	98.4	2022	[70]
22	EfficientNet	Differentiate NHL	CNN	95.6	2021	[44]
23	LymphoML	Predict lymphoma types	ML, LightGBM	85	2023	[50]
24	ImmunoGenius	Predict and differentiate lymphoma subtypes	ML, decision tree algorithm	91.8	2023	[52]

AI, artificial intelligence; AIPSS-MF, artificial intelligence prognostic scoring system for myelofibrosis; ALL, acute lymphocytic leukemia; AML, acute myelocytic leukemia; ANN, artificial neural network; BMT, bone marrow trephine; CLL-MRD, chronic lymphocytic leukemia minimal residual disease; CNN, convolution neural network; DL, deep learning; DNN, deep neural networks; ET, essential thrombocythemia; GBM, gradient-boosting framework; MF, myelofibrosis; NA, not applicable; PV, polycythemia vera; RBC, red blood cells; SVM, support vector machine; WSI, whole-slide imaging; XAI, explainable artificial intelligence; XGBoost, extreme gradient boosting.

## Data Availability

Not applicable.

## Abbreviations

2D	Two-dimentional
AA	Aplastic anemia
AI	Artificial intelligence
AIPSS-MF	Artificial intelligence prognostic scoring system for myelofibrosis
ALL	Acute lymphoblastic leukemia
ALPODS	Algorithmic population description approach
AML	Acute myeloid leukemia
ANN	Artificial neural network
APL	Acute promyelocytic leukemia
AUC	Area under the curve
BCB	Brilliant cresyl blue
BERMUDA	Batch effect removal using deep autoencoder
BMA	Bone marrow aspirate
BMS	Bone marrow smear
B-NHL	B- cell non-Hodgkin lymphoma
BNN	Bayesian neural network
CBC	Complete blood count
CD	Cluster of differentiation
CLL	Chronic lymphocytic leukemia
CML	Chronic myeloid leukemia
CNN	Convolution neural network
CNV	Copy number variation
CoAtNet	Convolution and attention network model
DNA	Deoxyribonucleic acid
DL	Deep learning
DLBCL	Diffuse large B-cell lymphoma
DMR	Differentially methylated region
DNN	Deep neural network
FCL	Follicular cell lymphoma
FDA	Food and Drug Administration
FDART	Federated gradient boosting trees with dropout
FGBT	Federated gradient boosting tree
FISH	Fluorescence in situ hybridization
FMLP	Federated multi-layer perceptron
FMNB	Federated multinomial naive bayes
H&E	Hematoxylin and eosin
Hb	Hemoglobin
Hb-H	Hemoglobin H
HPLC	High-performance liquid chromatography
IHC	Immunohistochemistry
LPL	Lymphoplasmacytic lymphoma
MALT	Mucosa associated lymphoma tissue
MCL	Mantle cell lymphoma
MDS	Myelodysplastic syndrome
MF	Myelofibrosis
MFC	Multiparameter flow cytometry
ML	Machine learning
MLP	Multi-layer perception
MM	Multiple myeloma
MOM	Multi-dimensional module optimization
MPN	Myeloproliferative neoplasm
MRD	Minimal residual disease
MulCNN	Multi-level convolutional neural network
MZL	Marginal zone lymphoma
PBMC	Peripheral blood mononuclear cell
PBS	Peripheral blood smear
PCT	Prediction confidence score
RBC	Red blood cell
RCA2	Reference component analysis 2
RGS1	Regulator of G-protein signaling 1
RNN	Recurrent neural network
ScRNA-seq	Single-cell RNA sequencing
SOM	Self-organized map
SVM	Support vector machine
WBC	White blood cell
WGS	Whole-genome sequencing
WSI	Whole-slide image
XAI	Explainable artificial intelligence
